# Predictive performances of 6 data mining techniques for obstructive sleep apnea-hypopnea syndrome

**DOI:** 10.1097/MD.0000000000029724

**Published:** 2022-06-30

**Authors:** Miao Luo, Yuan Feng, Jingying Luo, XiaoLin Li, JianFang Han, Taoping Li

**Affiliations:** a Department of Respiratory Medicine, Hospital Affiliated Guilin Medical College, Guilin, China; b Sleep Disorder Center, Nanfang Hospital, Southern Medical University, Guangzhou, China; c Department of Dermatology, The Second Affiliated Hospital of Guilin Medical University, Guilin, China.

**Keywords:** artificial neural network, Bayesian network, data mining, decision tree, prediction model, support vector

## Abstract

This study compared the effects of 6 types of obstructive sleep apnea-hypopnea syndrome (OSAHS) prediction models to develop a reference for selecting OSAHS data mining tools in clinical practice.

**Methods:**

This cross-sectional study included 401 cases. They were randomly divided into 2 groups: training (70%) and testing (30%). Logistic regression, a Bayesian network, an artificial neural network, a support vector learning machine, C5.0, and a classification and regression tree were each adopted to establish 6 prediction models. After training, the 6 models were used to test the remaining samples and calculate the correct and error rates of each model.

**Results:**

Twenty-one input variables for which the difference between the patient and nonpatient groups was statistically significant were considered. The models found the abdominal circumference, neck circumference, and nocturia ≥2 per night to be the most important variables. The support vector machine, neural network, and C5.0 models performed better than the classification and regression tree, Bayesian network, and logistic regression models.

**Conclusions:**

In terms of predicting the risk of OSAHS, the support vector machine, neural network, and C5.0 were superior to the classification and regression tree, Bayesian network, and logistic regression models. However, such results were obtained based on the data of a single center, so they need to be further validated by other institutions.

## 1. Introduction

Obstructive sleep apnea-hypopnea syndrome (OSAHS) is a common disease that afflicts approximately 4% of Chinese adults^[1–10]^ and 2%–5% of all people worldwide.^[11–13]^

With the growing number of OSAHS patients that have been diagnosed at sleep centers, many hospitals have accumulated considerable amounts of data on them. More efficient and sophisticated theoretical methods and tools are required in order to take advantage of the rich amount of data. The discovery of unknown and valuable information as well as patterns from big data has been made possible by advances in computer performance and artificial intelligence and the development of machine learning theory. Data mining techniques make it possible to extract information and knowledge even without a distinct hypothesis. This is achieved by exploring and analyzing a large amount of data based on research objectives with automation tools. There have been many successful applications of data mining techniques in the medical field. Data mining techniques have been used for the diagnosis^[14–20]^ and screening^[21]^ of OSAHS patients, prognosis of the efficacy and compliance of continuous positive airway pressure treatment,^[22–24]^ and determining the correlation between OSAHS and complications.^[25]^

OSAHS may result from the interplay between genetic factors and other risk factors such as obesity and smoking. OSAHS patients often exhibit symptoms such as snoring, a lack of energy, and nocturia. Because the neck circumference (NC), chest circumference (CC), abdominal circumference (AC), and body mass index (BMI) of OSAHS patients are higher than average, there have been many reports on using demographic and physical indicators to predict the risk of OSAHS. Conventional statistical methods generally make use of logistic regression (LR) models. These prediction models can help in identifying high-risk patients for OSAHS but still do not provide a satisfactory performance. Comparative analyses on the abilities of different data mining techniques to predict the risk of OSAHS have been rare. In this study, we used demographic, symptomatic, and physical indicators to compare the performances of OSAHS prediction models constructed according to six different methods: LR, support vector machine (SVM), artificial neuron network (ANN), C5.0, classification and regression tree (CRT), and Bayesian method. Our aim was to provide a reference for the selection of clinical screening tools for high-risk OSAHS patients.

## 2. Methods

### 2.1. Patients

The information was retrieved from the OSAHS database at Southern Hospital of Southern Medical University. This study was approved by the Medical Ethics Committee of the Southern Hospital. From September 2009 to June 2011, the subjects of this cross-sectional study were enrolled from Sleep Center, Nanfang Hospital, Southern Medical University. The medical records of 401 patients who had undergone PSG due to sleepiness, snoring, or other symptoms suspicious for OSAHS were reviewed. In total, 401 cases of patients who underwent a complete polysomnography as well as the demographic, symptomatic, and physical indicators were included in the analysis. The OSAHS diagnostic criteria included snoring, sleepiness, apnea, and an apnea/hypopnea index of ≥5.

### 2.2. Variable inclusion methods

Univariate analysis was performed on the demographic, symptomatic, and physical indicators. In total, the indicators that showed *P* < 0.05 in the univariate analysis or those with important clinical significance were used as the input variables for the data mining. Cases with and without OSAHS were used as the output variables.

### 2.3. Statistical and analytical methods

The data management and analysis was performed by using the Statistical Product and Service Solutions 13.0 and Clementine 12.0 software. The data of the 401 cases were randomly divided into training (70%) and testing (30%) datasets with a random seed of 4016978. This resulted in 284 cases in the training dataset and 117 cases in the testing dataset. We used 6 types of data mining techniques to construct the models: LR, Bayesian networks, ANNs, SVM, C5.0, and CRT. After training with the 284 cases in the training dataset, the 6 constructed models were used to assess the 117 cases of the testing dataset. The testing dataset was used to calculate the correct and error rates of each model. The areas under the receiver operator characteristic (ROC) curves of the 6 models were compared. The chi-square test was used to examine the categorical variables, whereas continuous variables were assessed with the Kolmogorov-Smirnov normality test. The normally distributed data were expressed as the mean ± standard deviation, and the independent samples *t* test was used to compare the two groups. Skewed distributions were represented by the median (interquartile range), and the Mann-Whitney *U* test was used to compare the groups. In our Bayesian model, the prior probability was 0.817, and the posterior probability was 0.821. The details of Bayesian model are as follows: Structure type: Markov Blanket; Including feature selection preprocessing; Parameter learning method: maximum likelihood ratio; Pattern: simple; Independent test: likelihood ratio; Significance level: 0.01; Maximum condition set size: 5.

## 3. Results

We selected 21 input variables that showed a significant difference between the OSAHS and non-OSAHS groups or those with important clinical significance: gender (i.e., whether or not the patient was male), age, course of disease, snoring, apnea, sleepiness during daytime, lack of energy, waking up early, dry mouth, nocturia ≥ twice per night, difficulty falling asleep, smoking, height, weight, NC, CC, AC, BMI, nighttime systolic blood pressure, morning systolic blood pressure, and morning diastolic blood pressure. Table 1 presents the basic features and distribution of these variables.

**Table 1:** Distribution of the input variables in the OSAHS and non-OSAHS groups.

Variables	OSAHS group (n = 328)	Non-OSAHS group (n = 73)	Statistics	*P* value
Male, n (%)	298 (91)	52 (71)	20.708	<0.001
Age	46 ± 11	41 ± 12	2.985	0.003
Course of disease (mo)	120 (60–120)	60 (34–120)	–3.798	<0.001
Snoring, n (%)	316 (96)	65 (89)	5.263	0.022
Hyperactivity, n (%)	36 (11)	8 (11)	0	0.997
Apnea, n (%)	253 (77)	39 (53)	16.958	<0.001
Daytime sleepiness, n (%)	145 (44)	41 (56)	3.433	0.064
Lack of energy, n (%)	216 (66)	19 (26)	39.039	<0.001
Dizziness, n (%)	142 (43)	34 (47)	0.261	0.609
Apathy, n (%)	12 (4)	1 (1)	0.401	0.527
Hearing loss, n (%)	4 (1)	1 (1)	0.011	1
Morning headache, n (%)	79 (24)	21 (29)	0.699	0.403
Memory loss, n (%)	94 (29)	23 (32)	0.234	0.628
Short attention span, n (%)	48 (15)	10 (14)	0.042	0.837
Waking up early, n (%)	5 (2)	8 (11)	14.069	<0.001
Chest tightness, n (%)	29 (9)	12 (16)	3.754	0.053
Dry mouth, n (%)	242 (74)	43 (59)	6.427	0.011
Mouth pain, n (%)	40 (12)	7 (10)	0.392	0.531
Belching, n (%)	2 (1)	0 (0)	0.806	1
Drooling, n (%)	2 (1)	0 (0)	0.806	1
Night sweat, n (%)	23 (7)	6 (8)	0.13	0.719
Nocturia ≥2 times, n (%)	115 (35)	11 (15)	11.076	0.001
Decreased libido, n (%)	5 (2)	1 (1)	0	1
Irritability, n (%)	2 (1)	1 (1)	0.395	0.454
Difficulty falling asleep, n (%)	17 (5)	10 (14)	5.606	0.018
Smoking, n (%)	138 (42)	17 (23)	8.886	0.003
Height (cm)	170 (165–172)	168 (162–170)	–2.593	0.01
Weight (kg)	79 (72–89)	68 (60–75)	–6.446	<0.001
NC (cm)	40 (38–42)	37 (35–40)	–6.684	<0.001
CC (cm)	101 (97–106)	94 (89–100)	–6.433	<0.001
AC (cm)	100 (95–107)	90 (85–98)	–7.124	<0.001
Nighttime systolic blood pressure	130 (120–138)	126 (116–135)	–1.926	0.054
Nighttime diastolic blood pressure	80 (75–86)	79 (73–86)	–1.205	0.228
Morning systolic blood pressure	129 (120–140)	122 (112–131)	–3.709	<0.001
Morning diastolic blood pressure	83 (77–90)	80 (72–85)	–3.312	0.001
BMI	28 (26–29)	25 (22–27)	–6.316	<0.001

AC = abdominal circumference, BMI = body mass index, CC = chest circumference, NC = neck circumference, OSAHS = Obstructive Sleep Apnea-Hypopnea Syndrome.

Table 2 presents the order of importance of the input variables in 5 models except the Bayesian network model as it does not contain this feature. The 5 most important variables that demonstrated the highest frequency were AC (5/5), neck circumference (3/5), and nocturia ≥ 2 times (3/5).

**Table 2:** Order of importance of the input variables in the 5 models.

Order of importance	Logistic regression	Support vector machines	C5.0	Artificial neural networks	Classification and regression tree
1	AC	NC	AC	Gender	AC
2	NC	Chest tightness	Nocturia ≥ 2 times	Age	NC
3	Age	Apnea	Body weight	Course of disease	Morning diastolic blood pressure
4	Sleepiness during daytime	AC	Morning systolic blood pressure	Snoring	Nocturia ≥ 2 times
5	Apnea	Nocturia ≥ 2 times	Course of disease	Apnea	Age

AC = abdominal circumference, NC = neck circumference.

OSAHS was present in 81% of the cases in the testing dataset. Table 3 presents the diagnostic performances of the 6 prediction models for the 177 cases in the testing dataset. The SVM model showed the highest accuracy.

**Table 3:** Comparison of the correct rates of the 6 prediction models.

Model	Correct rate (n)	Error rate (n)	Total
Logistic regression	84.62% (99)	15.38% (18)	100% (117)
ANN	88.03% (103)	11.97% (14)	100% (117)
CRT	84.62% (99)	15.38% (18)	100% (117)
SVM	88.89% (104)	11.11% (13)	100% (117)
C5.0	88.03% (103)	11.97% (14)	100% (117)
Bayesian network	81.20% (95)	18.80% (22)	100% (117)

ANN = artificial neural network, CRT = classification and regression tree, SVM = support vector machine.

**Figure 1.**
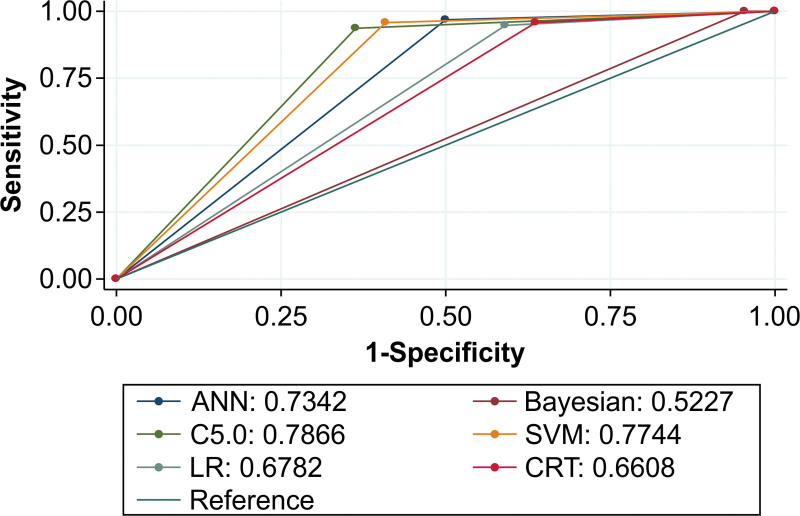
Areas under the ROC curves of the 6 models (*P* = 0.0001). ANN = artificial neural network, CRT = classification and regression tree, LR = logistic regression, ROC = receiver operator characteristic, SVM = support vector machine.

**Figure 2.**
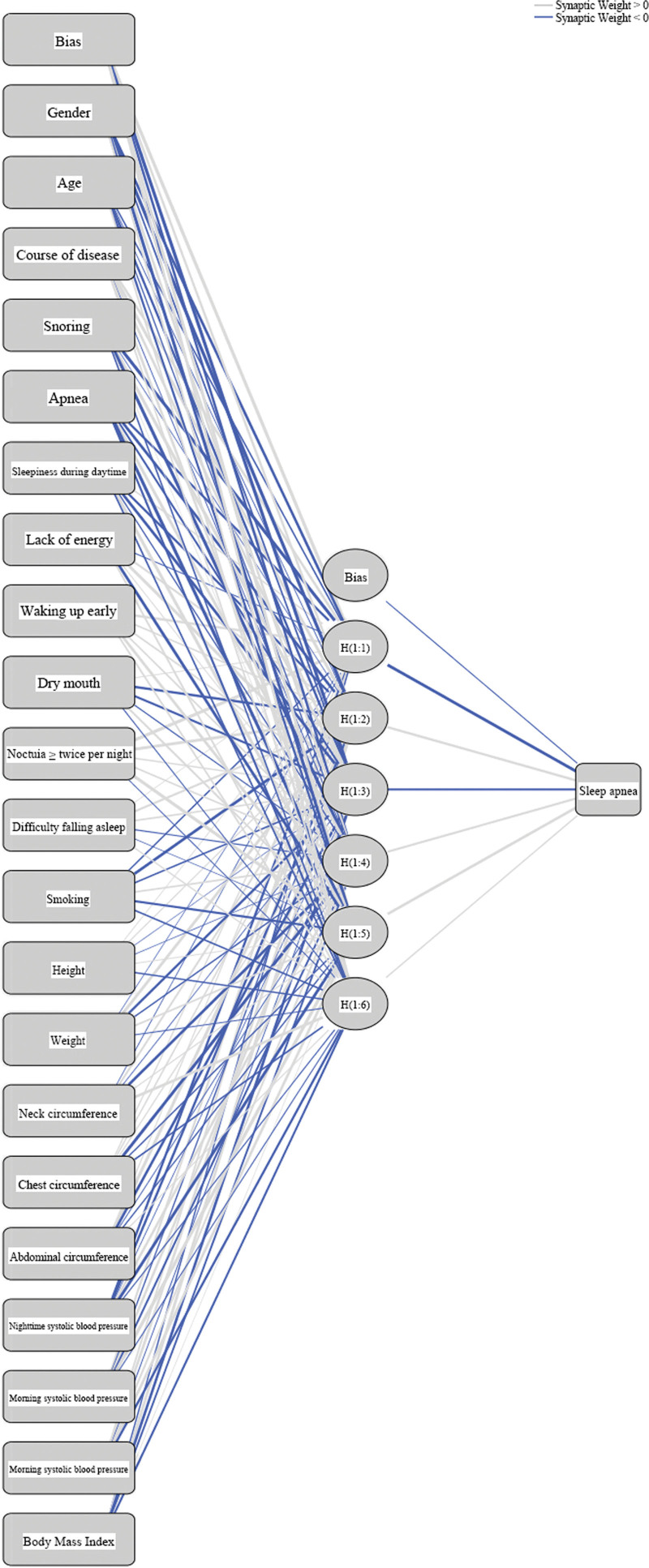
The neural network graph.

The results from comparing the areas under the ROC curves of the 6 models were consistent with the correct rates. Figure 1 shows that the C5.0, SVM, and neural network models had the largest areas, whereas the LR, CRT, and Bayesian models had the smallest areas. Figure 2 is the graphical representation of the neural network model.

## 4. Discussion

Our results suggest that the SVM, ANN, and C5.0 methods predicted OSAHS better than the CRT, Bayesian network, and conventional LR methods in the high-risk group of patients who sought treatment at the sleep centers. Among the symptomatic and physical indicators of OSAHS patients, the AC, NC, and nocturia ≥ 2 times were found to be the most important risk factors of OSAHS.

Not much research is available on the diagnostic significance of clinical symptomatic and physical indicators for OSAHS. This is mainly due to the low specificity of a single symptom or physical indicator. However, we believe that the probability of illness can be fairly accurately predicted before a polysomnography by combining multiple symptomatic and physical indicators. We believe that these indicators are not inferior in accuracy to currently available tests, which are costly and invasive. These indicators are easily available and require only an integration tool for application.

We found that AC and NC are among the most important predictors of OSAHS, which is consistent with the results of Caffo et al.^[21]^ Because we did not include the snoring frequency and snoring sound intensity, we were unable to determine the importance of snoring characteristics to predicting OSAHS. We also found that nocturia of more than twice per night is one of the most important risk factors for OSAHS. Nocturia is related to decreased renin-angiotensin-aldesterone secretion, increased atrial natriuretic peptide release, exaggerated intrathoracic pressure swings, and disturbed sleep. Increased frequency of nocturia in OSAHS patients has been demonstrated in several studies.^[26–29]^

Our results indicated that the SVM, ANN, and C5.0 models provided a better predictive performance than the conventional LR and Bayesian network models. These results differ from those of Marcos et al,^[16]^ who found that the Bayesian network achieved the best performance. We speculate that the difference between the results may be related to the inclusion of different variables and different software processing. Although the performance was not ideal, Eiseman et al^[17]^ still believed that clinical and electrocardiographic characteristics could be applied to predicting apnea with a certain level of accuracy. We believe that the predictive performance relies not only on the model construction methods but also on the variables included in the analysis. The results showed that our SVM model performed better than that reported by Eiseman et al.^[17]^ We speculate that this may be related to the differences in the variables included in the analysis. El-Solh et al^[18]^ included subjective indicators in addition to the age, BMI, and NC as input variables, which made their results incompatible to ours. However, we believe that, although clinical symptoms are important, some difficult-to-change variables, such as the course of disease, smoking, gender, and AC, are more important.

The LR model performed poorly compared with several modeling techniques for unknown reasons. Although LR is a classic modeling approach, several other data mining algorithms that were used in our study and were developed in recent years have been widely used in many fields and outperformed the conventional LR method.^[30–33]^ This shows that current modeling techniques that rely solely on conventional statistical methods may not be able to obtain the best results. In addition to developments in LR methods, OSAHS researchers should also determine new modeling algorithms, and the advantages and disadvantages of various statistical methods should be assessed after their practical applications are compared.

By using data mining techniques, we may identify patterns in data and discover new knowledge without a prior hypothesis. Data mining is a powerful tool to extract useful information from vast amounts of clinical data. Decision trees, Bayesian networks, ANNs, and SVM learning are among several types of relatively mature methods. These methods had certain differences in the selection of the order of importance of the OSAHS predictors, which reflects the difference between different modeling algorithms. However, these methods agreed that the AC, NC, and nocturia are all primary risk factors. This is also consistent with impressions obtained from routine clinical practice, which strengthens the notion that these three variables are indeed the most important risk factors for OSAHS.

Different data analysis methods involve different theories and algorithms. For clinical practice, the key is which methods produce results that are closest to actual observations. The construction of a model can be considered as a scientific hypothesis that requires further testing to determine its consistency with reality. In order to avoid overestimating the accuracy of the model, independent samples that are different from samples used to construct the model are needed to verify the model. External independent data are also required to assess the applicability of the model. The limitation of our study was that our data were derived from a single sleep center; patients from different geographical regions may be more heterogenic, so the conclusions from other studies may differ from ours. Therefore, our conclusions still need to be verified by other research groups.

In conclusion, our study showed that, in terms of OSAHS risk prognosis, the SVM learning, ANN, and C5.0 models outperformed the LR, CRT, and Bayesian network models. Because our conclusion was derived from the data of a single sleep center, the information may not be representative, and our results should be verified by other research groups, our findings are not applicable to the general population.

## Author contributions

Conceptualization: Jingying Luo, Miao Luo,Taoping Li.

Data curation: XiaoLin Li, JianFang Han,.

Methodology: Miao Luo, Yuan Feng, Jingying Luo.

Software: Yuan Feng.

Supervision: Taoping Li, Yuan Feng.

Writing – original draft: Miao Luo.

Writing – review & editing: Taoping Li.
